# Hypoxia Inducible Factor-2α (HIF-2α) Pathway Inhibitors

**DOI:** 10.15586/jkc.v12i3.413

**Published:** 2025-07-02

**Authors:** Ratika Dogra, Ulka Vaishampayan

**Affiliations:** 1 Hospitalist, Internal Medicine, Mercy St Vincent Hospital, Toledo, OH, USA;; 2 Professor of Medicine/Oncology, University of Michigan, Ann Arbor, MI, USA

**Keywords:** belzutifan, hypoxia, hypoxia inducible factors, LITESPARK, von Hippel–Lindau

## Abstract

Hypoxia creates a stressful environment for the cells triggering adaptive changes in the transcription factors called hypoxia inducible factors (HIF), which help to meet the metabolic and angiogenic requirements of cells. HIF-2 is one such factor that is implicated in the progression of various cancers, especially the ones associated with Von Hippel–Lindau (VHL) disease. HIF-2 factor has an unstable component alpha and a stable component beta. HIF-2α detects hypoxia and dimerizes with HIF-beta (Aryl hydrocarbon receptor nuclear translocator [ARNT]) through per-ARNT-sim (PAS)-mediated signaling domains. These domain sites are recognized as targets for HIF-2 inhibitors. HIF-2 inhibitors block this heterodimerization and prevent the expression of target genes which are oncogenic, including *VEGF, PDGF, CAIX*, and *Oct4*. VHL disease caused by the deficiency of *VHL* gene product results in decreased degradation of HIF-2α, leading to increased activation of these transcription factors. Tumors driven by the deficiency of *VHL* gene product are natural candidates for HIF-2 inhibitor therapy. These inhibitors have emerged as a promising class of targeted therapies for renal cell carcinoma (RCC), particularly in cases resistant to conventional treatments. In this review, we explore the role of hypoxia and HIF transcription factors in tumor formation and progression, highlighting the role and development of HIF-2 pathway inhibitors as potential cancer therapies. We discuss the major key inhibitors, with focus on belzutifan and review the various trials investigating its efficacy in monotherapy as well as in combination therapies in RCC. Additionally, we explore its development in pheochromocytoma, hemangioblastoma, and pancreatic neuroendocrine tumors. We also highlight emerging HIF-2α inhibitors currently in clinical trials, namely casdatifan, NKT-2152, and DFF332. Finally, we address the major toxicities and management of these inhibitors.

## Introduction

### Cellular response to hypoxia

Hypoxia is one of the stimuli for cells to increase angiogenesis, cell proliferation. Tumor cells are actively dividing at increased rate, causing an increased need for oxygen. Within the low oxygenated tumor microenvironment, tumor cells thrive by triggering metabolic and angiogenic switches through prominent activation of a class of transcription factors, the hypoxia-inducible factors (HIF) (1). The HIF-regulated cascades operate at moderate to weak hypoxia (<1% O_2_), and the unfolded protein response (UPR) activated by endoplasmic reticulum (ER) operates at more severe hypoxia (<0.2%) (2). Gene-expression profiling analysis in distinct cell types revealed tissue- and cell-specific variations in hypoxia response, although a consensus hypoxia response signature was shown to be a general poor-prognosis indicator for diverse cancer types (3). Hypoxia contributes to many critical aspects of cancer, including genome instability, autophagy, metabolic reprogramming, angiogenesis, migration, invasion, extracellular matrix remodeling, epithelial mesenchymal transition (EMT), stem cell maintenance, immune evasion, and therapy resistance (4). Angiogenesis in response to hypoxia is a part of adaptive response, increasing oxygen and nutrient delivery to tissues. This is mediated by pro-angiogenic factors, such as vascular endothelial growth factor (VEGF). Tumor cells decrease the level of anti-angiogenetic factors, increasing the angiogenesis and improving the oxygen delivery (5). Hypoxia activates the expression of *SNAIL, TWIST1, TCF3, ZEB1*, and *ZEB2* to promote EMT of tumor cells (6). The transition of epithelial cells to mesenchymal cell is an important aspect of cell differentiation and development that helps with healing and stem cell behavior but can go to other end of spectrum and can cause fibrosis and cancer progression. These changes are finely regulated at transcriptional, translational, and post-translational levels in the cell (7). During EMT, epithelial cells lose their junctions and apical–basal polarity, reorganize their cytoskeleton, and undergo a change in signaling programs that define cell shape and reprogram gene expression, which then increases the motility of individual cells and enables the development of an invasive phenotype (8, 9). The mesenchymal conversion helps cells to migrate and invade into surrounding tissues (10).

### Structure of HIF

The key regulator of hypoxia-induced transcriptional changes in all metazoans, also known as “master regulator,” is, the heterodimeric HIF that belongs to the family of transcription factors (11, 12). The HIF family has three different isoforms. While HIF-1 and HIF-2 are known transcription activators, HIF-3 is considered a negative regulator of hypoxia response pathway (13). HIF-1α and HIF-2α are closely related, as both activate hypoxia response element (HRE)-dependent gene transcription (14). HIF-3α is a more distantly related isoform than HIF-1α and HIF-2α, and in certain splicing arrangements, it encodes a polypeptide that antagonizes HRE-dependent gene expression (15). HIF-1 is the only transcription factor noted to be active under hypoxia. It consists of 120-kD HIF-1α subunit and a 91–94-kD HIF-1β subunit (16). The beta subunit is a subtype of mammalian aryl hydrocarbon receptor complex, which is a common subunit of HIF family, such as HIF-1, HIF-2, and HIF-3 (12). Alpha subunit is unstable whereas beta subunit is stable.

Hypoxia inducible factor is usually stabilized by a small reduction of the pericellular oxygen concentration from about 4% (which is a normal level in most normal tissues) to about 1% O_2_, that is, from ∼40 to ∼10 µM. Such reduction is readily experienced in various normal tissues under certain conditions, for example, in muscles during physical exercise (17). Alpha detects hypoxia and attaches to beta and forms a transcription complex with specific locations at DNA called HRE, which induces hypoxia inducible gene transcription and causes angiogenesis, cell proliferation, and erythropoiesis (18). Activation of alpha subunit is frequently involved in cancers. Under normal oxygen conditions, HIFα subunits are rapidly degraded by proteasome through a ubiquitin complex comprising von Hippel–Lindau (VHL) protein, elongins B and C, and Cullin 2 (CUL2) (19). VHL attaches to alpha subunit and prevents it from joining beta subunit, which then prevents excessive angiogenesis. This is helpful in tumor cancers with high angiogenesis (20). HIF-1α protein acts as a tumor suppressor and is frequently lost through inactivating mutations, 14q chromosome deletions, HIF-2α acts as an oncogene promoting the expression of its target genes (*VEGF, PDGF, CAIX Oct4*, among others) (21). In addition to hypoxia, the activity of HIF-1α is also co-regulated by two signal pathways—the phosphatidylinositol-3-kinase–protein kinase B–mammalian target of sirolimus (PI-3K/Akt/mTOR) pathway and the extracellular-regulated kinase–mitogen-activated protein kinase (ERK/MAPK) pathway (22).

HIF-2α is strongly expressed in well-vascularized areas. HIF-2α protein was stabilized at 5% O_2_ (resembling end-capillary oxygen conditions) in contrast to low HIF-1α activity at this oxygen level, actively transcribing genes such as *VEGF*. Under hypoxia (1% O_2_), HIF-1α was transiently stabilized and primarily mediated acute responses whereas HIF-2α protein gradually accumulated and governed prolonged hypoxic gene activation (23). HIF-2 consists of HIF-2α (EPAS 1) and HIF-β (ARNT), where two components heterodimerize with each other. Although this complex involves protein–protein interactions mediated by basic helix–loop–helix and per-ARNT-sim (PAS) domains in both proteins, the role played by PAS domains is poorly understood (24).

The *VHL* gene product, pVHL, is a multifunctional protein that shuttles between the nucleus and cytoplasm (25). The VHL protein is a component of substrate recognition pocket of E3 ubiquitin ligase responsible for ubiquitination and proteasome-mediated degradation of HIF-α subunits (21). Under hypoxia, HIF-1α is not hydroxylated and is released from *VHL*-mediated degradation. Stabilized HIF-1α moves into the nucleus, dimerizes with HIF-1β, and binds to HRE (26). Loss of *VHL* function leads to increased expression and stabilization of hypoxia inducible factors (HIF) leading to increased activation of hypoxia-related response elements. VHL is caused by germline loss of function of the *VHL* gene on one allele at chromosome 3p25-26. A somatic “second hit” event leads to the loss of other allele and tumor formation (27).

## von Hippel–Lindau (VHL) disease

von Hippel–Lindau disease is hereditary autosomal-dominant tumor in which carriers of disease-causing germline variant in *VHL* gene are at increased risk of developing benign and malignant tumors in different organs (28, 29). Mutations in the *VHL* tumor suppressor gene located on chromosome 3 causes VHL disease. In all, 500 different pathogenic germline mutations have been identified in families with VHL disease (30). VHL syndrome is characterized by hemangioblastomas of the brain, spinal cord, and retina; renal cysts and clear cell renal cell carcinoma (ccRCC); pheochromocytoma and paraganglioma; pancreatic cysts and neuroendocrine tumors; endolymphatic sac tumors; and epididymal and broad ligament cystadenomas (31).

von Hippel–Lindau disease is highly penetrated and almost 100% patients are affected by age 60. The incidence is 1 in 36,000 live births, and its penetrance is up to 90% (32). The clinical suspicion of *VHL* gene mutation warrants gene counseling for gene mutation screening. The most common tumors are benign hemangioblastoma, RCC, pheochromocytoma, pancreatic neuroendocrine tumor (pNETs), endolymphatic sac tumors, cysts in kidney, pancreas, uterine ligament. There are two types of VHL disease. Type 1 VHL is predominantly associated with large deletion or truncation mutations, which result in an encoded protein with very little or no activity. It is associated with retinal and central nervous system (CNS) hemangioblastoma and RCC but not pheochromocytoma. Type 2 VHL is usually associated with missense mutations encoding a protein with limited activity and includes pheochromocytoma with or without other clinical findings (29).

VHL can be classified as follows:

- Type 1 (without pheochromocytoma)
- Type 2 (with pheochromocytoma). Type 2 is further classified as:
- Type 2A: Pheochromocytoma is present along with CNS hemangioblastomas but no RCC.
- Type 2B: Pheochromocytoma is present along with both CNS hemangioblastomas and RCC.
- Type 2C: Pheochromocytoma is present without hemangioblastomas or RCC.

## HIF-2α Inhibitors

As a transcription factor, HIF-2α has long been considered non-targetable, as transcription factors mostly do not contain active ligand-binding sites amenable to inhibition via small molecules; however, it was discovered that a domain is present in HIF-2α, which enables protein–protein interaction required for the assembly of a functional HIF complex, which could be blocked by a small-molecule inhibitor (24). A study conducted by Erbel et al. demonstrated that HIF-2α PAS-B binds the analogous ARNT domain *in vitro*, showing that residues involved in this interaction are located on the solvent-exposed side of the HIF-2α central β-sheet (33). Mutating residues at this surface disrupts the interaction between isolated PAS domains *in vitro* and interferes with the ability of full-length HIF to respond to hypoxia in living cells. Extending these findings to other PAS domains, it was noted that β-sheet interface is widely used for both intra and intermolecular interactions, suggesting a basis of specificity and regulation of many types of PAS-containing signaling proteins (33). Tumors driven by deficiency of *VHL* gene product are natural candidates for HIF-2 inhibitor therapy. HIF-2α overexpression is ubiquitous in VHL disease-associated RCC and is associated with sensitivity to HIF-2α inhibitor therapy in xenograft models (34). HIF-2α subunit antagonists block HIF pathway activation at its most proximal source. A structural analysis at the University of Texas Southwestern Medical Center identified vulnerability in the alpha subunit, which heterodimerizes with HIF-1β, ultimately leading to the development of PT2385, a first-in-class inhibitor (35).

## HIF-2α inhibitors in RCC

About 70% of patients with VHL disease develop RCC by 60 years of age and it is the leading cause of death of these patients (36). The diseased VHL kidney is characterized by renal cysts and clear cell carcinoma. HIF-2α is always over expressed in ccRCC; however, 40% of ccRCC is unable to produce HIF-1α due to deletion of the *HIF-1*α gene situated in the 14q region in combination with inactivating mutations (37, 38).

## First-generation HIF-2α inhibitors

In the initial development of HIF-2α inhibitors, two potential drugs were developed, PT2399 and PT2385. A study conducted by Chen et al. studied PT2399 *in vivo*, showing activity in both tumor cell lines and patient-derived xenografts, in both treatment-naïve and Sunitinib-resistant tumors, and was associated with a reduction in the levels of circulating erythropoietin, a target of HIF-2α in blood (39). PT2399 had greater activity than sunitinib, was active in sunitinib-progressing tumors, and was better tolerated. Unexpectedly, some *VHL*-mutant ccRCCs were resistant to PT2399. Resistance occurred despite HIF-2 dissociation in tumors and evidence of HIF-2 inhibition in mouse, as determined by suppression of circulating erythropoietin, HIF-2 target, and the possible pharmacodynamic marker. Sensitive tumors exhibited a distinguishing gene expression signature and generally higher levels of HIF-2α. Prolonged PT2399 treatment led to resistance noted to be by binding site and second site suppressor mutations in HIF-2α and HIF-1β, respectively. Both mutations preserved HIF-2 dimers despite treatment with PT2399. Finally, an extensively pretreated patient whose tumor had given rise to a sensitive tumor graft showed disease control for more than 11 months when treated with a close analogue of PT2399 and PT2385 (39).

PT2385 was also validated in multiple *in vivo* models, showing dramatic tumor responses in animal models and consistent dose-dependent inhibition of HIF-2α targets, while no effect was observed in genes that are targets of HIF-1α (40). Unfortunately, *in vivo* studies showed a wide range of bioavailability with a significant number of patients not being able to achieve the targeted 500-ng/mL concentration of the drug in the plasma (41). Phase I dose-escalation trial of PT2385 in 51 patients with previously treated advanced ccRCC by Courtney et al. enrolled 26 patients in the dose-escalation portion and 25 in the expansion portion of the study (42). All patients were previously treated with VEGF-targeted therapy. No dose-limiting toxicity was observed with any dose. Based on safety, pharmacokinetic, and pharmacodynamic profiling, the recommended Phase II dose (RP2D) was defined as 800 mg twice per day. PT2385 was well tolerated, with anemia (grade 1–2, 35%; grade 3, 10%), peripheral edema (grade 1–2, 37%; grade 3, 2%), and fatigue (grade 1–2, 37%; no grade 3 or 4) being the most common treatment-emergent adverse events. Complete response, partial response, and stable disease as best response were achieved by 2%, 12%, and 52% of patients, respectively (42).

## Second-generation HIF-2α inhibitors

Based on the findings of Phase 1 trials of the first-generation HIF-2α inhibitors, second-generation of HIF-2α inhibitors were developed with improved pharmacokinetic profiles, selectivity, and potency. Most studied drug in this category is belzutifan (also called MK-6482 and PT2977). Other newer drugs include casdatifan, DFF332, and NKT2152, which are currently under trials to study efficacy (43).

Belzutifan (MK-6482, previously called PT2977), a highly specific and well-tolerated HIF-2α inhibitor, received approval from the US Food and Drug Administration (FDA) on August 13, 2021 for the treatment of VHL disease patients requiring therapy for associated nonmetastatic RCC, pNETs, and CNS hemangioblastomas and carrying *VHL* germline mutations (26, 44, 45). Belzutifan binds to the PAS-B pocket of HIF-2α and destabilizes the dimerization of HIF-2α and ARNT through allosteric effects, mainly mediated by the key residue M252 of HIF-2α near the dimer interface (46).

The first-in-human Phase 1 study of belzutifan (NCT02974738) was performed by Choueiri et al., where patients had advanced solid tumors (dose-escalation cohort) or previously treated advanced ccRCC (dose-expansion cohort) (47). In all, 95 patients were enrolled. Belzutifan was administered orally using a 3+3 dose-escalation design, followed by expansion at RP2D in patients with ccRCC. In the dose-escalation cohort (*n* = 43), no dose-limiting toxicities occurred at doses of up to 160 mg once daily (OD), and the maximum tolerated dose was not reached; the RP2D was 120 mg once daily. Plasma erythropoietin reductions were observed at all doses; erythropoietin concentrations correlated with plasma concentrations of belzutifan. In patients with ccRCC who received 120 mg once daily (*n* = 55), the confirmed objective response rate (ORR) was 25% (all partial responses), and the median progression-free survival (PFS) was 14.5 months. The most common grade ≥3 adverse events were anemia (27%) and hypoxia (16%). Belzutifan was well tolerated and demonstrated preliminary anti-tumor activity in heavily pre-treated patients, suggesting that HIF-2α inhibition might offer an effective treatment for ccRCC (47). As observed after a median follow-up of >3 years for patients still receiving treatment in Phase 1 LITESPARK-001 (MK-6482-001) study of belzutifan in advanced solid tumors, belzutifan monotherapy continued to show a high rate of disease control and durable responses in previously treated patients with advanced ccRCC. Belzutifan exhibited a favorable safety profile, and no new safety signals were observed (48).

The NCT04846920, Phase 1, dose-escalation study evaluated the safety and tolerability of belzutifan (MK-6482) in participants with advanced ccRCC (49).

Jonasch et al. performed a Phase 2, open-label, single-group trial of 61 patients with RCC associated with VHL disease with measurable, localized RCCs of <3 cm; the researchers investigated the efficacy and safety of HIF-2α inhibitor belzutifan (NCT03401788) administered orally at a dose of 120 mg daily (50). The primary endpoint was objective response (complete or partial response). In all, 49% of patients with RCC associated with VHL disease who received belzutifan had a confirmed objective response; most patients had a reduction in renal tumor size. Responses were also observed in patients with pancreatic lesions (47 of the 61 patients [77%]) and CNS hemangioblastomas (15 of the 50 patients [30%]). Among the 16 eyes that were evaluated in 12 patients with retinal hemangioblastomas at baseline, all (100%) were graded as showing improvement. The most common adverse events were anemia (in 90% of patients) and fatigue (in 66% of patients) (50). After a median follow-up of 29.3 months, belzutifan continued to show antitumor activity in VHL disease-related neoplasms, including RCC, pNETs, and CNS hemangioblastomas, whereas the safety profile remained consistent with that of the previous reports. These results support the use of belzutifan as a systemic treatment for VHL disease and it had received FDA approval for *VHL* indication (51). It is approved for adult patients with VHL disease who require therapy for associated RCC, CNS hemangioblastomas, or pNET that do not require immediate surgery. Further extended follow-up with more than 2 years of data in RCCs associated with VHL disease who received belzutifan reported an ORR of 59% in this trial (51).

A Phase 3 LITESPARK-005 (NCT04195750), an open-label, randomized, head-to-head trial was performed with 746 patients having unresectable locally advanced or metastatic ccRCC that had progressed following a programmed death receptor-1 (PD-1) or programmed death-ligand 1 (PD-L1) checkpoint inhibitor and a VEGF–tyrosine kinase inhibitor (TKI). Patients were randomized 1:1 to receive 120-mg belzutifan or 10-mg everolimus once daily. In all, 374 participants were randomized to belzutifan and 372 to everolimus. On December 14, 2023, the US FDA approved belzutifan for patients with advanced RCC, following a PD-1 or PD-L1 inhibitor and VEGF-TKI, following the findings of this trial. The major efficacy outcome measures were PFS assessed by blinded independent central review and the overall survival (OS). A statistically significant improvement in PFS was demonstrated for belzutifan, compared to everolimus, with a hazard ratio of 0.75 ([95% confidence interval CI: 0.63, 0.90]; 1-sided P = 0.0008). Kaplan–Meier curves reflected non-proportional hazards with similar median PFS estimates of 5.6 months (95% CI: 3.9, 7.0) in the belzutifan arm and 5.6 months (95% CI: 4.8, 5.8) for those receiving everolimus. While OS results were immature at the current analysis, with 59% of deaths reported, no trend toward a detriment was observed. Patient-reported symptom and functional outcomes were supportive of improved tolerability for belzutifan, compared to everolimus. At stage IA1 (median follow-up: 18.4 months), PFS and ORR were superior with belzutifan versus everolimus, and OS was not statistically significant. Compared to everolimus, belzutifan was associated with less worsening of disease-related symptoms of kidney cancer and better preservation of health-related quality of life (HRQOL). At stage IA2 (median follow-up: 25.7 months), PFS, OS, and ORR results were consistent with stage IA1. Complete responses occurred in 13 belzutifan (3.^5^%) versus 0 everolimus participants. More patients remained progression-free with belzutifan versus everolimus at 12 months (PFS 33.7% [belzutifan] vs. 17.6% [everolimus]) and 18 months (PFS 22.5% [belzutifan] vs. 9.0% [everolimus]); 22.6% versus 5.0% of participants had ongoing treatment: 5.9% versus 14.7% of participants discontinued study therapy because of any adverse event. Grade 3–5 treatment-related adverse events (TRAE) occurred in 38.7% versus 39.4% of participants. The most common adverse reactions (≥25% incidence) in patients receiving belzutifan were decreased hemoglobin, fatigue, musculoskeletal pain, increased creatinine, decreased lymphocytes, increased alanine aminotransferase, decreased sodium, increased potassium, and increased aspartate aminotransferase (52, 53).

The LITESPARK-013 trial (NCT04489771), a Phase 2 trial, compared two belzutifan doses (120 mg daily vs. 200 mg daily) in patients with sporadic mRCC who had received up to three lines of prior therapy. The study showed that there was no significant difference in the primary endpoint of ORR as well as PFS and OS between either dose strengths. The safety profiles of the belzutifan 200 mg and 120-mg once daily doses in the LITESPARK-013 study were generally similar and consistent with the known safety profile of belzutifan. However, the belzutifan 200-mg once daily dose was associated with a higher proportion of the overall dose modification and drug discontinuation. These results, together with the results from the Phase 3 LITESPARK-005 study, continue to support the belzutifan 120-mg OD dose in patients with ccRCC (54, 55).

With increasing development and emerging data in RCC, HIF-2α inhibitors are also increasingly studied in sporadic RCC as well as in combination therapies. On December 14, 2023, the FDA approved belzutifan (Welireg, Merck & Co. Inc.) for patients with advanced RCC, following a PD-1 or PD-L1 checkpoint inhibitor and a VEGF-TKI based on the findings of LITESPARK 005 trial (52).

## Combination Therapies with Belzutifan

An open label Phase 2 study, LITESPARK-003, by Choueiri et al. studied belzutifan plus cabozantinib for patients with advanced ccRCC. Cohort 1 was treatment naïve patients and cohort 2 included patients with prior immunotherapy and up to two prior systemic therapies. Patients were given belzutifan 120 mg orally once daily and cabozantinib 60 mg orally once daily until disease progression, unacceptable toxicity, or patient withdrawal. The primary endpoint was investigator-determined ORR as per the Response Evaluation Criteria in Solid Tumors version 1.1 (RECIST v1.1), with secondary outcomes of PFS, duration of response (DOR), time-to-response (TTR), OS, and safety/tolerability (41, 56). The updated results presented in 2025 American Society of Clinical Oncology (ASCO) showed durable antitumor activity in both frontline and subsequent-line treatment of patients with RCC and a safety profile consistent with prior reports. In all, 50 patients were enrolled and treated in cohort 1 and 52 patients in cohort 2. Confirmed ORR was 70% (95% CI, 55–82; 6 complete responses [CRs], 29 partial responses [PRs]) in cohort 1 and 31% (95% CI, 19–45; 2 CRs, 14 PRs) in cohort 2. In cohort 2, ORR was 32% (95% CI, 16–52; 1 CR, 8 PRs) in patients who received prior immunotherapy only (n = 28) and 29% (95% CI, 13–51; 1 CR, 6 PRs) in patients who received both prior immunotherapy and anti-VEGFR-TKIs (n = 24). ORR by the International Metastatic Renal Cell Carcinoma Database Consortium (IMDC) risk and baseline tumor burden subgroups is shown in Table 1. Median DOR was 29.1 months (range: 1.9+ to 47.4; + indicates ongoing response at last assessment) in cohort 1 and 30.4 months (range, 4.2+ to 45.6) in cohort 2. An estimated 62% of responders in cohort 1 and 52% in cohort 2 remained in response for ≥24 months. Median PFS was 30.3 months (95% CI, 19.4–not reached) in cohort 1 and 13.8 months (95% CI, 9.2–19.4) in cohort 2. Median OS was not reached in cohort 1 and was 26.7 months (95% CI, 20.0–41.1) in cohort 2. Overall, 27 (54%) patients in cohort 1 and 34 (65%) patients in cohort 2 had a grade 3 or higher TRAE. No patient died due to a TRAE in cohort 1 but 1 patient (2%) died due to treatment-related respiratory failure in cohort 2 (57).

**Table 1:** Summary of recent trials of belzutifan as monotherapy.

Trial name and treatment details	Sample size	Treatment-related adverse events (TRAE), grade ≥ 3	Efficacy/study endpoints
LITESPARK 001 (NCT02974738) Phase 1 Belzutifan oral 3+3 dose escalation design, followed by expansion at RP2D in patients with ccRCC. 55 received 120 mg daily dose of RP2D	95	Anemia – 27% Hypoxia – 16%	ORR – 25% all partial responders (PR) Median PFS 14.5 months
LITESPARK 004 (NCT03401788) Phase 2 Belzutifan at a dose of 120 mg	61	Anemia – 7% Hypoxia – 1%	ORR – 59% in RCC Most patients had a reduction in renal tumor size
LITESPARK 018 (NCT04846920) Phase 1 Belzutifan monotherapy	29	Anemia – 48% Hypoxia – 31%	ORR – 7%
LITESPARK 005 (NCT04195750) Phase 3 belzutifan 120 mg vs. everolimus 10 mg once daily	746	Anemia – 29% Hypoxia – 10%	Median PFS – 5.6 months for both belzutifan and everolimus ORR – 22% for belzutifan vs. 4% for everolimus Median OS – 21.4 months for belzutifan vs. 18.2 months for everolimus
LITESPARK 013 (NCT04489771) Phase 2 Belzutifan 120 mg vs. 200 mg daily	154	Anemia – 200 mg: 26.9%, 120 mg: 19.7% Hypoxia 200 mg 21.8%, 120 mg 21.1%	The study showed that there was no significant difference in the primary endpoint of ORR as well as PFS and OS between either dose strengths ORR was 23.7% vs. 23.1% for the 120-mg and 200-mg groups

Phase 1/2 KEYMAKER-U03 Substudy 03B (NCT04626518) trial is conducted to evaluate combination treatments for previously treated advanced ccRCC. Results for targeted therapy containing regimens from arm B4 (pembrolizumab + belzutifan [HIF-2α inhibitor]), arm B5 (lenvatinib [VEGF-TKI] + belzutifan), and the reference (Ref) arm (pembrolizumab + lenvatinib) were presented. Lenvatinib + belzutifan (arm B5) exhibited durable antitumor activity and a safety profile consistent with the individual profiles of the drugs. Results from the Substudy-03B trial supported further investigation of lenvatinib + belzutifan combination for participants with advanced RCC, as in the LITESPARK-011 trial (58).

The Phase 3 study LITESPARK 011, studying belzutifan plus lenvatinib versus cabozantinib in advanced RCC after anti-PD-1/PD-L1 therapy, has completed accrual and results are awaited. This study was developed to evaluate efficacy and safety of belzutifan plus TKI lenvatinib versus TKI cabozantinib in patients with advanced RCC progressing after anti-PD-1/PD-L1 therapy in the first- or second line setting or as an adjuvant therapy. Approximately 708 patients were randomly assigned 1:1 to receive either 120-mg oral belzutifan plus 20-mg oral lenvatinib once daily or 60-mg oral cabozantinib once daily. The dual primary endpoints are PFS as determined by blinded independent central review per RECIST 1.1, and by OS; secondary endpoints include ORR, DOR, safety, and tolerability (59).

The LITESPARK-024 (NCT05468697) is an open-label, multicenter, Phase 1/2 randomized study of belzutifan + palbociclib (CDK 4/6 inhibitor) versus belzutifan monotherapy in patients with advanced RCC. Patients must have histologically confirmed unresectable stage IV RCC with a clear cell component; have received at least two prior systemic regimens (both an anti-PD-1/PD-L1 monoclonal antibody and a VEGF receptor-targeted TKI, in sequence or in combination); have measurable disease per RECIST v1.1 by blinded independent central review (BICR); have a Karnofsky Performance Status (KPS) score of ≥70%; and have radiographic disease progression on or after the most recent regimen per investigator (60). The primary endpoint of the Phase 2 portion is ORR with key secondary endpoints of OS, PFS, and safety (55, 60).

The LITESPARK-022 (NCT05239728) multicenter, double-blind, randomized, Phase 3 study is designed to compare the efficacy and safety of belzutifan + pembrolizumab with that of placebo + pembrolizumab as adjuvant treatment of ccRCC after nephrectomy. Approximately 1600 patients with histologically or cytologically confirmed RCC (intermediate high [pT2, grade 4 or sarcomatoid, N0, M0 or pT3, any grade, N0, M0], high [pT4, any grade, N0, M0 or pT, any stage/grade, N+, M0], or M1 NED [patients who present with the primary kidney tumor and solid, isolated, soft tissue metastases that can be resected at the time of nephrectomy or ≤2 years from nephrectomy]) with a clear cell component and had not previously received systemic therapy will be enrolled. Patients must have undergone nephrectomy and/or metastasectomy ≤12 weeks before randomization and must be tumor-free. The primary endpoint is disease-free survival (DFS), and key secondary endpoint is OS. Other secondary endpoints are safety, disease recurrence-specific survival, and patient-reported outcomes (61).

An open-label, randomized, Phase 3 study (NCT04736706) will compare first-line treatment with the novel combination therapies pembrolizumab + belzutifan + lenvatinib (arm A) or MK-1308A (pembrolozumab with quavonlimab) + lenvatinib (arm B) with pembrolizumab + lenvatinib (arm C) for advanced RCC. Dual primary endpoints are PFS and OS for arm A or arm B versus arm C in patients with IMDC intermediate/poor status and in all patients regardless of IMDC status. Secondary endpoints are ORR and DOR, patient-reported outcomes, and safety (62).

## Novel HIF-2 inhibitors

### Casdatifan (AB521)

HIF-2α inhibitor AB521 under clinical development by Arcus Biosciences and currently in Phase I trial for RCC targets and allosterically binds to a hydrophobic pocket on HIF-2α, leading to a confirmational change that prevents HIF-2α heterodimerization with HIF-1β and binding to the HRE binding site on DNA, resulting in decreased transcription and expression of HIF-2α downstream target genes, many of which regulate tumor cell growth and survival (63, 64). ARC-20A (NCT05536141), Phase 1 dose-escalation and dose-expansion study, is to investigate the safety, tolerability, and pharmacology of HIF-2α inhibitor AB521 monotherapy in patients with ccRCC and other solid tumors. Primary endpoints are the incidence of dose-limiting toxicity and adverse events. Secondary endpoints include ORR, AB521 plasma concentration, and AB521 pharmacokinetics (65). Various combination therapies are currently studied with AB521, including the combination therapy with cabozantinib and zanzalintinib (65–67).

### NKT2152

NKT2152 is a novel, potent, and selective orally available HIF2α inhibitor that has demonstrated robust activity in both ccRCC cell line-derived and patient-derived xenograft RCC and other solid tumor models. Phase 1/2, open label dose-escalation and expansion trial of NKT2152 is currently underway as a single agent administered orally once daily. The primary objective of Phase 1 is to determine the recommended dose for expansion and Phase 2 is ORR. Key secondary objectives include safety, tolerability, PD effects, PFS, DOR, and disease control rate. Exploratory objectives include evaluation of biomarkers predictive of tumor response (68, 69). A Phase 2 trial to evaluate the safety and efficacy of NKT2152 in combination with palbociclib (doublet) and with palbociclib and sasanlimab (triplet) in subjects with advanced or metastatic ccRCC is currently underway (70).

### DFF332

DFF332, a small molecule inhibitor that selectively targets HIF2α transcriptional activity, has shown dose-dependent antitumor efficacy in preclinical models of ccRCC. This is the first in human, Phase I/Ib, open-label, multicenter, study (CDFF332A12101, NCT04895748) of DFF332 in adult patients with advanced ccRCC. The study evaluated the safety, tolerability, antitumor activity, pharmacokinetics (PK) and pharmacodynamics of DFF332 and has shown a promising safety profile across all doses and schedules in Phase 1 study. Additionally, there have been indications of clinical activity and a dose-proportional modulation of erythropoietin (71).

**Table 2:** Belzutifan-based combination therapy trials for RCC.

Trial name and treatment details	Sample size	Efficacy/primary endpoints
LITESPARK-003 Phase 2 Belzutifan 120 mg orally once daily and cabozantinib 60 mg orally once daily Cohort 1 – treatment naïve Cohort2 – prior immunotherapy and up to two prior systemic therapies	Cohort 1 – 50 Cohort 2 – 52	Cohort 1 ORR – 70% Median PFS – 30.3 months 12 months OS – 96% 24 months OS – 86% Cohort 2 ORR – 31% Median PFS – 13.8 months
NCT04626518 KEYMAKER-U03 substudy 03B Phase 1/2 Arm B4 Pembrolizumab + belzutifan Arm B5 Lenvatinib + belzutifan Reference arm Pembrolizumab + lenvatinib	Arm B4 – 62 Arm B5 – 64 Reference arm – 73	Arm B4 ORR – 19.4% Median PFS – 5.4 months Arm B5 ORR – 46.9% Median PFS –12.5 months Reference arm ORR – 39.7% Median PFS – 9.4 months
LITESPARK-011 Phase 3 Belzutifan plus lenvatinib vs. cabozantinib monotherapy in patients with ccRCC with prior anti-PD-1/PD-L1 therapy	708	Ongoing trial, results are pending. Dual primary endpoints – PFS and OS
LITESPARK-024 (NCT05468697) Phase 1/2 Belzutifan + palbociclib vs. belzutifan monotherapy Phase 1 ≤30 participants will be enrolled into 3 dose groups and receive belzutifan 120 mg once daily + palbociclib (75, 100, or 125 mg) daily for 21 consecutive days, followed by 7 days off. Phase 2 Approximately 150 participants will be randomly assigned 2:1 to receive belzutifan 120 mg once daily + palbociclib RP2D (21 consecutive days/7 days off) or belzutifan 120 mg once daily	Phase 1 – ≤30 Phase 2 – 150	Primary endpoint of Phase 2 portion – ORR
LITESPARK-022 (NCT05239728) Phase 3 Adjuvant post-nephrectomy Belzutifan + pembrolizumab vs. pembrolizumab + placebo	1600 approx.	Disease-free survival (DFS)
NCT04736706 Phase 3 Metastatic front line Arm A Pembrolizumab + belzutifan + lenvatinib Arm B MK-1308A (pembrolozumab with quavonlimab) + lenvatinib Arm C Pembrolizumab + lenvatinib (arm C)	1431	Dual primary endpoints are PFS and OS for arm A or arm B vs. arm C in patients with IMDC intermediate/poor risk, and in all patients regardless of IMDC status

**Table 3:** Summary of ongoing trials of novel HIF-2 inhibitors.

Trial name and study design	Patient eligibility	Key endpoint/s
ARC 20 (NCT05536141) Phase 1/1b Two Stages evaluating AB521 – casdatifan dose and safety Stage 1 – Dose escalation Stage 2 – Dose expansion	Confirmed ccRCC who have received previous treatment for metastasis with an anti-programmed cell death protein 1 (anti-PD-1) therapy and a VEGF-targeting TKI	Primary endpoints – dose-limiting toxicity and adverse events Secondary endpoints – ORR, AB521 plasma concentration, and AB521 pharmacokinetics
XL092-009 (STELLAR-009) Phase 1b/2 Zanzalintinib (XL092) + AB521 vs. Zanzalintinib + AB521 + nivolumab	Participants with unresctable ccRCC or other advanced solid tumors with no prior treatment with HIF-2α-targeted therapies	Primary endpoints – recommended doses, safety and tolerability, pharmacokinetics, and measuring preliminary efficacy
NKT 2152-101 (NCT05119335) Phase 1/2 NK2152 Phase 1 – Dose escalation Phase 2 – evaluate the safety, pharmacokinetics, and antitumor efficacy of NKT2152	Patients aged 18 years or older with ccRCC who have exhausted available standard therapy as determined by the investigator	Primary objective – Phase 1 – determine the recommended dose Phase 2 – ORR Secondary objectives – safety, tolerability, PD effects, PFS, DOR, and disease control rate
NKT2152 (NCT05935748) Phase 2 Doublet therapy NKT2152 + palbociclib vs. Triplet therapy NKT2152 + palbociclib + sasanlimab	Patients with advanced or metastatic ccRCC who have received at least one prior therapy	Primary objective – ORR, dose-limiting toxicity Secondary objective – PFS, OS, pharmacokinetic profile
CDFF332A12101 (NCT04895748) Phase 1/1b Monotherapy DFF332 single agent Combination therapy DFF332 + everolimus or DFF332 + spartalizumab + taminadenant	Patients with advanced or metastatic ccRCC with progression after prior PD-1/L1 checkpoint inhibitor and VEGF-targeted therapy	Primary endpoint – safety and tolerability Secondary endpoint – antitumor activity and pharmacokinetics
PEAK-1 Phase 3 Arm A – casdatifan + cabozantinib vs. Arm B – cabozantinib monotherapy	Unresectable, locally advanced, metastatic ccRCC with progression following prior anti-PD-1, PD-L1 with no prior exposure to HIF-2αinhibitors or cabozantinib	Primary endpoint – PFS Secondary endpoint – OS

## Toxicity of HIF-2 Inhibitors

HIF-2 inhibitors have unique toxicities particularly related to their role in hypoxia response and erythropoiesis suppression. Anemia, hypoxia, hypertension are few of the common adverse reactions. The most-studied belzutifan has mainly low-grade adverse reactions that affect most patients using it (48, 50). Anemia is one of the common adverse reactions, especially in the first few weeks of the treatment and is related to the on-target effect on the HIF2α-target gene *erythropoietin* (*EPO*). Management includes supportive care with blood transfusion with erythropoietin-stimulating agents. Hypoxia is another adverse reactions related to HIF inhibitors. HIF is critical for cellular adaptation to low oxygen levels. HIF inhibition leads to hypoxia-related symptoms, such as dyspnea and dizziness. Regular pulse oximetry checks are very helpful for timely diagnosis. Most patients do well with supportive care; however, severe hypoxia may sometimes necessitate dose adjustment or discontinuation of the drug (26, 50). Fatigue is another common adverse reaction, possibly linked to anemia and metabolic changes with drug therapy. Appropriate support, good nutrition, hydration, and addressing anemia and hypoxia can help to manage fatigue symptoms (42, 48). Musculoskeletal pain, GI adverse reactions, such as nausea and vomiting, increased creatinine, decreased lymphocytes, increased alanine aminotransferase, decreased sodium, increased potassium, and increased aspartate aminotransferase are additional adverse reactions noted in the LITESPARK-005 study (52, 53). When used with immune checkpoint inhibitors or tyrosine kinase inhibitors, overlapping toxicities require closer patient surveillance. Belzutifan can render some hormonal contraceptives ineffective and its exposure during pregnancy can cause embryo-fetal harm (44).

### Hemangioblastoma

Capillary hemangioblastoma is a benign highly vascular tumor of CNS usually located in the cerebellum. It is observed to have a stromal component and capillary network. There is increasing evidence that the stromal cell population may represent the neoplastic component of hemangioblastoma whereas the vascular component may be composed of reactive, non-neoplastic cells (72). The stromal component of hemangioblastomas contains genetic alterations consistent with a neoplastic nature and studied to have loss of heterozygosity (LOH) of *VHL* gene locus and associated microsatellite regions (73). The stromal cells expressed abundant epidermal growth factor receptor (EGFR), and some stromal cells expressed platelet-derived growth factor receptors PDGFR-α, but not PDGFR-β. In contrast, endothelial cells coexpressed PDGFR-α and PDGFR-β. VEGF and placental growth factor (P1GF) were expressed by scattered stromal cells; however, more intense staining was observed in the endothelial cells of intratumoral blood vessels, possibly indicating the secreted growth factors bound to their target receptors (74). Overall, CNS hemangioblastomas occur in 60–80% of VHL patients (75). After the initial reports of response in the NCT03401788 Phase 2 trial (50), the early cases of using belzutifan in patients with hemangioblastoma showed significant reduction in the amount of perilesional edema, tumor diameter, and contrast enhancement just after three cycles (76).

### Pheochromocytoma

Pheochromocytomas and paragangliomas are associated with multiple hereditary disorders. The histologic features are distinguished from other VHL disease-associated clear cell neoplasms by more variable, frequently polyhedral cytology, basophilic and finely granulated cytoplasm, and cytoplasmic bodies in both familial and non-familial cases (77). These tumors are characterized by a thick vascular tumor capsule; myxoid and hyalinized stroma; round, small-to-medium tumor cells intermixed with small vessels; predominantly amphophilic and clear cytoplasm; absence of cytoplasmic hyaline globules; and lack of nuclear atypia or mitoses (78). HIF-2α is preferentially upregulated in *VHL*-mutant pheochromocytomas and paragangliomas (79).

The Pacak–Zhuang syndrome is a rare tumor-predisposition syndrome caused by gain-of-function mutations in the gene encoding HIF-2α (*EPAS1*) (80). Persons with this syndrome have polycythemia at an early age, with the subsequent development of multiple, recurrent, and occasionally metastatic paragangliomas that predominantly produce norepinephrine (81). Activating mutations in *EPAS1* lead to decreased degradation of HIF2α and consequent transcriptional up-regulation of hypoxia-related genes, including the gene encoding erythropoietin (*EPO*) (80, 82). The use of belzutifan, a potent and selective small-molecule inhibitor of the protein HIF-2α, was noted in a patient with polycythemia and multiple paragangliomas (the Pacak–Zhuang syndrome). The syndrome in this patient was caused by somatic mosaicism for an activating mutation in *EPAS1*. Treatment with belzutifan led to a rapid and sustained tumor response along with resolution of hypertension, headaches, and long-standing polycythemia (83).

### Pancreatic Neuroendocrine Tumors

Multiple, nonfunctional pNETs occur in VHL patients. Stromal collagen bands and clear-cell morphology are important histological features of VHL-associated NETs. The presence of allelic deletions of the *VHL* gene in pNETs provides direct molecular evidence for a role of the gene in their tumorigenesis and establishes NET as an independent tumor type of VHL disease (84). Multiple cysts and microcystic (serous) cystadenomas of the pancreas have also been reported occasionally in patients afflicted with VHL syndrome (85). All 61 patients in the Phase 2, open-label trial performed by Jonasch et al. had pancreatic lesions (50); a confirmed response was observed in 47 patients (77%), including 6 patients (10%) who showed complete response to belzutifan. Among 22 patients with pNETs, 20 patients (91%) had a confirmed response (including 3 patients [14%] who had a complete response) (50). A current Phase 2 study is ongoing to evaluate the efficacy and safety of monotherapy in participants with advanced pheochromocytoma/paraganglioma (PPGL), pNET, VHL disease-associated tumors, advanced gastrointestinal stromal tumor (wt GIST), or advanced solid tumors with HIF-2α-related genetic alterations (86).

## Conclusion

von Hippel–Lindau is a hereditary autosomal dominant disease responsible for the development of benign and malignant tumors. Awareness and testing of this disease for early detection and surgical management of the manifestations is critical. Close surveillance for multiple systems highlights the importance of multidisciplinary management. The advent of HIF-2α inhibitors has provided systemic therapy options for VHL as well as a novel therapeutic pathway that was proven to be effective in sporadic ccRCC. The future looks promising for VHL, and continued research and discovery, translational clinical trials, and comprehensive multidisciplinary care are critical to achieve optimal outcomes.
